# Editorial: Advances in diagnostics and interventions for acute aortic syndromes involving the aortic arch

**DOI:** 10.3389/fcvm.2026.1809419

**Published:** 2026-03-06

**Authors:** Thierry Carrel

**Affiliations:** Department of Cardiac Surgery, University Hospital Basel, Basel, Switzerland

**Keywords:** aortic (rupture) dissection, complication, endovascular, stent implantation, surgery

Acute aortic syndrome (AAS) is a group of life-threatening conditions that includes aortic dissection, intramural hematoma, and penetrating aortic ulcer. These entities share a similar clinical presentation, which is most commonly sudden, severe chest or back pain, and require rapid diagnosis and management. Despite recent advances in imaging and treatment, AAS remains associated with high morbidity and mortality. Current challenges include delayed recognition due to non-specific symptoms, overlap with other acute cardiovascular conditions, and still limited awareness in emergency settings. Timely access to high-quality imaging and multidisciplinary expertise in specialized centers is not always available. In addition, optimal patient selection for medical, endovascular, or surgical treatment remains complex, particularly when the involvement extends into the aortic arch (due to the proximity to cerebral vessels), and in elderly or high-risk patients.

Nevertheless, advancements in imaging technologies, biomarker science, surgical techniques, and endovascular interventions during the last decade have transformed how clinicians approach these disorders, improving diagnostic accuracy and expanding therapeutic strategies with the goal of reducing mortality and morbidity.

In the present Research topic, special attention has been given to two contributions on the “frozen elephant trunk” technique, a hybrid (surgical-endovascular) modification of the original surgical technique described by Borst several decades ago. This technique has allowed for some simplifications at the level of the distal anastomosis and may facilitate distal therapeutic extension using endovascular techniques.

One article emphasized the unique challenges of malperfusion syndrome and shed light on the potential of endovascular reperfusion to prevent the disastrous consequences of organ ischemia, followed by surgical repair, for those patients stable enough to delay ascending aorta repair. The final study presented an innovative, albeit complex, method of laser *in situ* fenestration used to connect the subclavian artery to the aortic graft in the setting of a hybrid arch debranching strategy.

For all the techniques presented here, centralizing patients at high-volume aortic centers with multidisciplinary teams may improve diagnostic turnaround times and outcomes.

In summary, the management of acute aortic syndromes involving the aortic arch includes medical stabilization, surgical repair, and increasingly, endovascular techniques. The choice of therapy depends, of course, on the aortic segments involved (Type A vs. Type B dissection), anatomical considerations, and patient stability. Initial medical therapy still focuses on pain control and blood pressure control to minimize shearing forces on the weakened aortic wall to allow for a safe transfer to a specialized center.

Open surgical reconstruction remains the gold standard for acute aortic syndromes but strategies to minimize or even eliminate hypothermic circulatory arrest have emerged in recent years and are now increasingly performed, as shown in this special issue. Endovascular technologies have also revolutionized the management of acute aortic syndromes, particularly in patients at high surgical risk or with favorable anatomy. While initially pioneered in descending aortic pathology (TEVAR), these techniques have been adapted to the challenging environment of the aortic arch. Techniques such as the *chimney*, *fenestration*, and *branched stent grafts* aim to preserve supra-aortic branch perfusion while excluding the diseased aortic segment. Custom fenestrated devices accommodate arch anatomy with arch branches, improving their suitability for endovascular repair. Hybrid interventions combine open debranching of cervical vessels with endovascular exclusion of the aortic arch.

Organizational advances, such as centralized *aortic centers* with dedicated multidisciplinary teams, will likely become more important for systematic improvements in time to diagnosis, quick implementation of newer and more complex procedures, and consolidation of early success and long-term survival. 

